# Sickle Cell Disease: Management Options and Challenges in Developing Countries

**DOI:** 10.4084/MJHID.2013.062

**Published:** 2013-11-04

**Authors:** Daniel Ansong, Alex Osei Akoto, Delaena Ocloo, Kwaku Ohene-Frempong

**Affiliations:** 1 School of Medical Sciences,-Kwame Nkrumah University of Science and Technology, Kumasi-Ghana; 2 Department of Child Health, Komfo Anokye Teaching Hospital, Kumasi-Ghana; 3 Children’s Hospital of Philadelphia, Director, Comprehensive Sickle Cell Centre, USA

## Abstract

Sickle Cell Disease (SCD) is the most common genetic disorder of haemoglobin in sub-Saharan Africa. This commentary focuses on the management options available and the challenges that health care professionals in developing countries face in caring for patients with SCD.

In a developing countries like Ghana, new-born screening is now about to be implemented on a national scale. Common and important morbidities associated with SCD are vaso-occlusive episodes, infections, Acute Chest Syndrome (ACS), Stroke and hip necrosis. Approaches to the management of these morbidities are far advanced in the developed countries. The differences in setting and resource limitations in developing countries bring challenges that have a major influence in management options in developing countries.

Obviously clinicians in developing countries face challenges in managing SCD patients. However understanding the disease, its progression, and instituting the appropriate preventive methods are paramount in its management. Emphasis should be placed on early counselling, new-born screening, anti-microbial prophylaxis, vaccination against infections, and training of healthcare workers, patients and caregivers. These interventions are affordable in developing countries.

## Introduction

Sickle Cell Disease (SCD) is the most common genetic disorder of haemoglobin in sub-Saharan Africa.^1^,^2^ In Africa it is estimated that about 200,000 children are born with the disease annually.^3^ A point mutation on the 6^th^ codon of the beta-globin gene located on the short arm of chromosome 11 produces a defective beta globin chain, which under low oxygen tension polymerizes into long fibres that eventually lead to abnormally deformed (sickled) red cell. The red cells thus become sticky, adhere to endothelium and clump together plugging micro-vessels^4^ and also damage large blood vessels that can become severely stenotic or occluded. In addition, sickle red cells have a much-shortened life span making chronic haemolytic anaemia a constant feature of SCD. The combination of vaso-occlusion and haemolytic anaemia contributes to the basic presentation of the disease and associated complications of which some are life threatening. Biomedical technology is moving very fast to use hematopoietic stem cell transplantation and gene therapy in the management of the disease in developed countries. However, only very few SCD patients have been successfully treated with hematopoietic stem cell transplantation^5^ and, gene therapy has not been successful yet in curing SCD.^6^ In general, the disease is managed using a combination of preventive and symptomatic therapies.^7^

This commentary focuses on the management options available and the challenges that health care professionals in developing countries face in caring for patients with SCD.

## Sickle Cell Vaso-Occlusive Episodes

The hallmark of SCD is the occurrence of vaso-occlusive episodes. These are characterized clinically by pain in the affected sites of the body. Pain episodes are largely unpredictable and but can be prevented and in more than 80% of the episodes, there is no potential precipitating cause.^8^ The pathophysiology of the pain is as a result of hypoxia and release of inflammatory mediators and pain neurotransmitters.^9^–^11^ Infections are thought to be playing a role in the pathogenesis of vaso-occlusive episodes,^12^ making the occurrence of vaso-occlusive episode a trigger to alerting potential problems in SCD. The severity of pain in people with SCD varies from mild to severe and the management is determined by accurate estimation of the severity and the associated pre-morbid conditions.^7^

### Management Options

The choice of an analgesic is of importance for effective pain management and so is the identification of the underlying cause that precipitated the occurrence of the episodes. The range of analgesics used to manage pain in SCD patients is similar in developed and developing countries. The analgesic spans from acetaminophen, non-steroidal anti-inflammatory drugs (NSAID) to opioids.^13^,^14^ There are several behavioural and non-medicinal therapies that are also available in the management of pain. There is evidence to show that hydroxyurea ameliorates the episodes of vaso-occlusive crisis in adults^15^,^16^ and in children in the recent BABY HUG study.^17^ Hydroxyurea can be used in SCD patient to achieve great benefit in preventing painful episodes. It is worthy of note that the use of hydroxyurea comes with caution because of the risk of neutropenia.

### Challenges

The long-term use of the analgesics comes with major challenges and complications. Patients develop gastric erosion and ulcers when they are placed on NSAIDs. In addition, the long-term use of these drugs can have effects on the kidneys. Clinicians are also confronted with side effect of the long-term use of opioids like pethidine, codeine and morphine in the management of pain without significantly causing respiratory depression with parenteral use and tolerance with long term use.^18^ Clinicians in developing countries should therefore use opioids with caution for the fear of respiratory depression as well as development of tolerance. The option of hydroxyurea to ameliorate the episodes of vaso-occlusive episode would need more research in the African population.

## Infections in Sickle Cell Disease

The risk of infection in SCD patient is well established because of several factors, namely, hyposplenism or asplenia and also, the availability of free iron radicals for growth of bacteria, defective chemotaxis and opsonisation, and presence of dead necrotic tissue in some parts of the body especially the bones.^4^,^11^ Children with SCD are particularly prone to infection by encapsulated organisms like *Strep pneumoniae*, *Haemophilus influenza B* and *Salmonella typhi*^19^–^21^ as well as intracellular organisms like *Mycoplasma pneumoniae*. Falciparum malaria is an important infection in SCD patients and contributes to the development of anaemia and hospitalization.^20^,^22^ Malnutrition is a risk factor for infection. Infections and the existence of malnutrition in African is a major concern. A study in Ghana revealed that the prevalence of malnutrition was 61.3% among SCD subjects and 28.6% among controls, (p<0.001).^23^ Infection predisposes the SCD patients to other events such as painful episodes, acute chest syndrome, sequestration episodes and hyper haemolytic episodes.

### Management Options

The management of infections in SCD patients does not solely depend on the isolated use of antibiotics but also on the recognition of other factors and effectively managing them promptly. In developing countries, the choice of antibiotics takes into consideration the universal principles for the use of antibiotics for bacterial infection. However local sensitivity pattern, cost and the disease severity are essential factors to consider. The most important interventions in preventing infections: provision of basic amenities like clean water, adequate sanitation facilities and appropriate nutritional education and counselling are required to significantly reduce the risk of acquiring infections in the general population as well as in SCD patients who are at increased risk of infections. Penicillin prophylaxis following new-born screening, and anti-pneumococcal vaccination are among the most significant life-saving preventive interventions in the management of children with SCD.^24^–^26^

### Challenges

The effective management of infections in SCD patient obviously depends on the availability of functional microbiology laboratories. Very good microbiology laboratory provides information on microbial type as well as sensitivity pattern. The lack of this facility in most health institutions in developing countries is a major challenge to the care of patients. The availability of the drugs play important role in making an appropriate choice. Clinicians in developing countries are sometimes limited in their choices.

Delays in treatment for bacterial infections and for children with malaria in endemic areas could lead to fatal consequences and therefore it is recommended that SCD children with febrile illness should have prompt diagnosis of malaria followed by its treatment and antibiotics immediately even if blood culture and sensitivity cannot be done. This approach will save more lives in areas lacking the resources and facilities.

## Acute Severe Anaemia

Acute severe anaemia is one of the common clinical presentations of SCD. The management of acute severe anaemia in these patients requires accurate diagnosis of the aetiology of the anaemia. The causes of anaemia in SCD patients are intra-vascular or extra-vascular haemolysis, sequestration into the spleen or the liver and transient red cell aplasia (“aplastic crisis”) usually following parvovirus B19 infection.^27^,^28^ The commonest predisposing factor contributing to increased haemolysis is infection. In addition, acute severe anaemia is also often related to acute chest syndrome.

### Management options

The modalities of treatment of anaemia in a SCD patient will largely depend on the cause of the anaemia. In correcting acute severe anaemia, packed red cells is the preferred choice of transfusion product and should be given cautiously and in small steps, allowing time for fluid equilibration, in order to avoid hypervolemia. Whole blood may be used where fractionation of blood is unavailable or in situations, such as acute splenic sequestration, where volume repletion may also be indicated. Underlying infections should be treated in all situations simultaneously.

Anti-malaria prophylaxis is currently not being encouraged in the routine care of SCD patients. There is a knowledge gap as to whether prophylaxis would be beneficial in the routine care given to SCD patients.^20^,^22^ Prompt diagnosis and early treatment of malaria as recommended by WHO Roll Back Malaria programme should remain the standard practice till more evidence become available.

Parents’ education related to spleen palpation and detection of sudden pallor or jaundice as well as observing urine colour for evidence of haemolysis is an extremely and inexpensive way to detect severe anaemia to prevent death.

### Challenges

Weak laboratory support in health institutions does not allow clinicians to make complete and confirmatory diagnosis of the causes of acute severe anaemia. Complete blood count can provide some useful information for the clinical care of the patient but does not sufficiently provide the clues for diagnosis. It is important to always determine the reticulocyte count in SCD patients. This broadly enables clinicians to categorize anaemia into aplastic crisis or as a result of increased haemolysis and or sequestration crisis.

Diagnosis of the cause of severe anaemia in SCD is based on history, physical examination and limited laboratory results in most hospital settings in sub-Sahara Africa. Lack of automated haematology analysers in laboratories to provide prompt complete blood count is a major concern since clinicians do not have enough clinical information to confirm diagnosis. In most laboratories reticulocyte counts are never done or results are unduly delayed and this puts clinicians in treatment dilemmas. Blood safety and availability are major challenges to clinicians. Blood transfusion is key to the management of people with SCD. Some institutions provide specialized blood-banking services to their SCD patients. Others would have to refer to centres with the blood transfusion facilities.

## Acute Chest Syndrome (ACS)

Acute chest syndrome is one of the life threatening complications of SCD that is associated with high mortality if not diagnosed promptly and managed with critical care and accuracy. ACS may be caused by vaso-occlusive damage of lung tissue, infection, or both, and replaces the term pneumonia because the possible causes for the two are not readily distinguishable. The classical definition of the condition is usually difficult to establish since it relies on radiological findings. However, all clinicians responsible for the care of SCD patients should have a high index of suspicion for the diagnosis of ACS in patients with chest pain and respiratory distress, otherwise appropriate treatment may be delayed, resulting in high mortality. Often patients develop ACS in the context of pain episodes being managed with increased hydration and parenteral opioid analgesics.

### Management Options

The treatment options include antibiotics, adequate pain control, hydration, careful monitoring for the need for and provision of supplementary oxygen, and blood transfusion.^29^ Hydration requires caution since over hydration has the potential of worsening the condition of the patient. ACS is a painful episode and therefore adequate analgesics should be provided with caution to avoid respiratory distress especially in instances where opioids have to be used. Broad-spectrum antibiotics, plus macrolides, are used in order to cover the possibility of bacterial infection, and especially mycoplasma and chlamydia, which are sometimes associated with ACS. The choice of antibiotics is critical and it is based on the issues raised in treatment of infections. Red cell transfusion is indicated based on degree of hypoxia, respiratory distress, and anaemia. Again clinical trials involving hydroxyurea has shown that the medicine can be used to ameliorate the episodes of ACS in adults and children.^15^–^17^

### Challenges

Radiological facilities in health institutions to support diagnosis are usually lacking. However radiological support in health institutions is not a crucial requirement to make a definitive diagnosis of ACS. The absence of this facility requires clinicians to apply their clinical judgement to treat ACS without radiological confirmation.

The absence of this facility requires clinicians to apply their clinical judgement to treat ACS without radiological confirmation. The distinction between pneumonia and pulmonary infarction in SCD is clinically immaterial since the management of ACS is designed to address both possibilities.

## Stroke

The diagnosis and management of stroke remains one of the biggest challenges facing clinicians in developing countries. Although stroke is a clinical diagnosis, brain imaging studies are necessary to distinguish infarctive from haemorrhagic stroke in order to apply correct intervention. Clinicians in developing counties should have a high level of suspicion of stroke in their SCD patients with sudden onset of neurological changes and offer them the best support. Often musculo-skeletal manifestations like limping or change in gait is attributed to vaso-occlusive episode in the SCD patient because it is the most frequent presentation. However a carefully history and examination may indicate the occurrence of stroke.

### Management and Challenges

Management of acute stroke includes vital signs monitoring and support, exchange red cell transfusion, and neurosurgical intervention in severe haemorrhagic stroke. Once stroke is diagnosed, the long-term management to prevent recurrence is a major challenge in developing countries. This is because the patient requires chronic transfusion therapy i.e. transfusion every 3–4 weeks and management of transfusion-related complications such as iron overload.^30^,^31^ There is currently no other option available for the management of SCD stroke as the recently the SWITCH study was ended prematurely because of safety concerns that favoured the use of blood transfusion with of iron chelating agents as compared to hydroxyurea with phlebotomy.^32^ Earlier a study had shown that hydroxyurea could reduce the Trans-Cranial Doppler flow rate in children.^33^

Stroke is more prevalent in children and its prevention has become a major effort in the management of SCD. Identification of the child with SCD at increased risk of stroke using Trans-Cranial Doppler (TCD) ultrasonography, followed by preventive chronic transfusion therapy, as practiced in developed countries,^34^ is non-existent in most developing countries. The offer of chronic blood transfusion is a major challenge in most African settings. Safe and reliable supply of blood is critical in the effective management of this complication in SCD patients. As many as 20% of children with SCD-SS with no history of overt stroke, have been found on brain Magnetic Resonance Imaging (MRI) to have “silent” cerebral infarcts.^35^ These infarcts predispose to higher risk for completed stroke and neurocognitive defects. Screening of children with SCD with brain MRI is not routinely available in developing countries.

## Hip Necrosis

The most common long-term orthopaedic complication seen in SCD in developing countries is aseptic necrosis of the head of the femur.^36^,^37^ The condition starts with an asymptomatic or localized mild hip pain and limping which progresses to severe hip joint deformity.^38^

### Management options

Individuals suffering from this complication living in developing countries are placed on long-term analgesics that only provide temporal relief of pain. The best options are physical therapy in milder cases or palliative surgical interventions, and ultimately hip replacement for advanced cases.^39^ However, very few hospitals have the capabilities to perform hip replacement surgery or core decompression due to several reasons including cost, required expertise and equipments. There are currently no preventive measures in place to prevent avascular necrosis of the femoral head; however the use of crutches is encouraged to the delay the need for replacement and also use of hydroxyurea has been associated with the reduction in incidence and progression.^40^ Hydroxyurea is also limited in developing countries because of limited laboratory facilities and client compliance factors. The overall benefit of the hydroxyurea however favours its used to address most of the complication of SCD. Patient and parental education is paramount in using the drug.

## Summary and Current Therapeutic Options

The current management strategies for SCD could be considered under the public health principles of early diagnosis, primary prevention and prompt management of acute episodes and complications.

The successes of early detection and prevention programmes in some North African countries should be emulated.^41^ Early diagnosis and primary prevention will constitute early detection of the disease coupled with counselling and use of Penicillin V and Folic acid.^42^ In areas where malaria is endemic, the use of treated mosquito bed-nets, indoor residual spraying and prompt treatment is highly recommended.

There are clear evidence in favour for the use of pneumococci vaccines in reducing morbidities and mortalities associated with SCD patients.^43^ In the United States of American SCD patients benefit from this preventive intervention. GAVI since 2009 has begun a programme of introducing new vaccines like pneumococci vaccine into the EPI systems of some sub-Saharan counties like Gambia (2009) Mali (2011) and Ghana, Tanzania (2012).^44^–^46^ The introduction of vaccines like Pneumococcal and Haemophilus vaccines protect SCD children against these highly virulent encapsulated organisms would have a great impact on the quality of life and survival of SCD patients. Hydroxyurea, a cytotoxic has been found to improve the clinical course of the disease. The incidence and severity of most of the complications of the disease are remarkably reduced under hydroxyurea therapy.^47^ It however requires careful monitoring in sub-Saharan Africa as limited evidence exists in patients in malaria endemic areas as well as the risk of other infections. Chronic blood transfusion is now indicated for SCD patients with higher than usual risk of stroke. However the risk of iron overload should be considered in all patients on the regimen.

Prompt diagnosis, identification of causal factors and management of the complications are key to the survival of the SCD patient. In developing countries diagnostic support required to compliment the clinical information is usually lacking and therefore clinicians are faced with challenges during the management of the disease. SCD is the most common genetic disorder in sub-Saharan Africa and organized specialized clinics would promote and facilitate the care of patients suffering from the disease. Furthermore, health information materials should be made available for health workers and patients. Lastly, as technology advances the need to fund more research into interventions such as transplantation should be encouraged.

## Conclusion

Obviously clinicians in developing countries face several challenges in managing SCD patients. However understanding the disease, its progression, and instituting the appropriate preventive methods are paramount in its management. Emphasis should be placed on new-born screening, anti-microbial prophylaxis, vaccination against infections, and training of healthcare workers, patients and caregivers. These are affordable in developing countries.

Efforts should be made to develop scientific research that would focus on solutions to improve on morbidity and mortality as well as quality of life
